# Parasail: SIMD C library for global, semi-global, and local pairwise sequence alignments

**DOI:** 10.1186/s12859-016-0930-z

**Published:** 2016-02-10

**Authors:** Jeff Daily

**Affiliations:** Pacific Northwest National Laboratory, High Performance Computing Group, 902 Battelle Boulevard, P.O. Box 999, MSIN J4-30, Richland, 99352 WA USA

**Keywords:** Smith-Waterman, Needleman-Wunsch, Semi-global alignment, Sequence alignment, SIMD, Database search

## Abstract

**Background:**

Sequence alignment algorithms are a key component of many bioinformatics applications.

Though various fast Smith-Waterman local sequence alignment implementations have been developed for x86 CPUs, most are embedded into larger database search tools. In addition, fast implementations of Needleman-Wunsch global sequence alignment and its semi-global variants are not as widespread. This article presents the first software library for local, global, and semi-global pairwise intra-sequence alignments and improves the performance of previous intra-sequence implementations.

**Results:**

A faster intra-sequence local pairwise alignment implementation is described and benchmarked, including new global and semi-global variants. Using a 375 residue query sequence a speed of 136 billion cell updates per second (GCUPS) was achieved on a dual Intel Xeon E5-2670 24-core processor system, the highest reported for an implementation based on Farrar’s ‘striped’ approach. Rognes’s SWIPE optimal database search application is still generally the fastest available at 1.2 to at best 2.4 times faster than Parasail for sequences shorter than 500 amino acids. However, Parasail was faster for longer sequences. For global alignments, Parasail’s prefix scan implementation is generally the fastest, faster even than Farrar’s ‘striped’ approach, however the opal library is faster for single-threaded applications. The software library is designed for 64 bit Linux, OS X, or Windows on processors with SSE2, SSE41, or AVX2. Source code is available from https://github.com/jeffdaily/parasail under the Battelle BSD-style license.

**Conclusions:**

Applications that require optimal alignment scores could benefit from the improved performance. For the first time, SIMD global, semi-global, and local alignments are available in a stand-alone C library.

**Electronic supplementary material:**

The online version of this article (doi:10.1186/s12859-016-0930-z) contains supplementary material, which is available to authorized users.

## Background

Sequence alignment is an order-preserving way to map characters between two DNA or amino-acid (protein) sequences. It is a pervasive operation in bioinformatics workflows used to identify regions of high similarity between sequences. Similarity is generally measured by assigning a positive score to matches and a negative score to mismatches. For proteins, a substitution matrix, such as BLOSUM [1] or PAM [2], is used to score amino acid similarity for each possible residue pair. In addition to negative scores, alignments may be penalized by the insertion of gaps or deletion of characters. Gap penalties are often linear (a fixed negative value per gap) or affine [3], where the gap opening penalty is typically larger than the gap extension penalty.

There are three primary classes of sequence alignment, namely global, semi-global, and local. A *global alignment* causes the alignment to span the entire length of each sequence and is used when the two sequences are similar in length and presumed to be related. A *local alignment* identifies highly conserved regions or subsequences though the rest of the sequence may be divergent. A *semi-global alignment* does not penalize beginning or end gaps in a global alignment such that the resulting alignment will tend to overlap one end of a sequence with an end of the other sequence.

Sequence alignments are computed using dynamic programming because it is guaranteed to find an optimal alignment given a particular scoring function. Regardless of the class of alignment being computed, a dynamic programming recurrence of the following form is computed. Given two sequences *s*_1_[ 1…*m*] and *s*_2_[ 1…*n*], three recurrences are defined as follows: Let *S*_*i,j*_ denote the optimal score for aligning the prefixes *s*_1_[ 1…*i*] and *s*_2_[ 1…*j*] such that the alignment ends by substituting *s*_1_[ *i*] with *s*_2_[*j*]. *D*_*i,j*_ denotes the optimal score for aligning the same two prefixes such that the alignment ends in a deletion, i.e., aligning *s*_1_[ *i*] with a gap character. Similarly, *I*_*i,j*_ denotes the optimal score for aligning the prefixes such that the alignment ends in an insertion, i.e., aligning *s*_2_[*j*] with a gap character. Given the above three ways to end an alignment, the optimal score for aligning the prefixes corresponding to the subproblem {*i,j*} is given by: *T*_*i,j*_=*max*(*S*_*i,j*_,*D*_*i,j*_,*I*_*i,j*_). The dependencies for the individual dynamic programming recurrences are as follows: *S*_*i,j*_ derives its value from the solution computed for the subproblem {*i*−1,*j*−1}, while *D*_*i,j*_ and *I*_*i,j*_ derive their values from the solutions computed for subproblems {*i*−1,*j*} and {*i,j*−1}, respectively.

A typical implementation of this dynamic programming algorithm builds a table of size $\mathcal {O}(m\times n)$ with the characters of each sequence laid out along one of the two dimensions. Each cell (*i,j*) in the table stores three values *S*_*i,j*_, *D*_*i,j*_, and *I*_*i,j*_, corresponding to the subproblem {*i,j*}. Given the dependencies of the entries at a cell, the dynamic programming algorithms for all three sequence alignment classes can be represented using the pseudocode outlined in Algorithm 1. The algorithm has a time complexity of $\mathcal {O}(mn)$.



The three classes of sequence alignment initialize the first row and column differently (lines 1 and 2 in Algorithm 1). SW and SG alignments initialize the first row and column of the table to zero, while NW alignments initialize the first row and column based on the gap function. The table values for SW alignments are not allowed to become negative, while NW and SG allow for negative scores.

Hereafter, for convenience according to common practice, we call the sequence with characters along the rows of the table the “query” sequence and the sequence with characters along the columns of the table the “database” sequence.

Aligning two sequences of lengths *m* and *n* requires $\mathcal {O}(mn)$ time. This computation time becomes much more significant when computing many alignments as done in many bioinformatics applications, such as database search, multiple sequence alignment, genome assembly, and short read mapping. There have been many approaches to making this operation faster including heuristic methods such as BLAST [4], however such heuristic methods may generate sub-optimal alignments.

There have been numerous efforts to parallelize optimal sequence alignments using vector instructions [5–12]. However, not all of these approaches necessarily address the same bioinformatics application. For example, database search may group database sequences to improve performance [8], while protein homology graph applications may prohibit such optimizations [13]. That said, parallel sequence alignments generally fall into two categories: inter-sequence and intra-sequence. Inter-sequence parallelization is the alignment of a single (query) sequence against a set of (database) sequences [8], while intra-sequence parallelization is focused on parallelizing a single pairwise alignment of two sequences. Zhao’s implementation of Farrar’s “striped” method [7, 9] was previously the fastest intra-sequence implementation of Smith-Waterman while Rognes’s SWIPE software is the fastest known inter-sequence implementation. No known implementations of vectorized Needleman-Wunsch or semi-global pairwise alignments existed for intra-sequence parallelism prior to the Parasail library. Two additional software libraries implement Rognes’s inter-sequence approach, opal (formally SWIMD) [14] and libssa [15]. Opal implements all classes of sequence alignments while libssa implements only local and global alignments. Both libraries support the SSE4.1 as well as the AVX2 instruction sets.

## Implementation

Parasail is a SIMD C (C99) library containing implementations of the Smith-Waterman (local), Needleman-Wunsch (global), and semi-global pairwise sequence alignment algorithms. Here, semi-global means insertions before the start or after the end of either the query or target sequence are not penalized. Parasail implements most known algorithms for vectorized pairwise sequence alignment, including diagonal [5], blocked [6], striped [7], and prefix scan [13]. In addition, the Parasail library implements each of these methods for the SSE2, SSE4.1, AVX2, and KNC (Xeon Phi accelerator) instruction sets. Parasail uses a technique called CPU dispatching to correctly select the appropriate implementation, at runtime, for the highest level of CPU instruction set supported. Therefore, Parasail is a reference implementation for these algorithms in addition to providing an implementation of the best-performing algorithm(s) to date on the most advanced CPUs. Source code is available at https://github.com/jeffdaily/parasail under the Battelle BSD License. The same files are included in a gzipped tar archive as Additional file 1.

### C library interface

There are over one thousand functions within the Parasail library. To make it easier to select a particular function, the function names follow a naming convention. The following will use parentheses “()” to indicate a selection from a set must be made. If a portion of the function name is optional, the description will indicate as such. Underscores “_” separate each function name component. The components of the function names are listed in the order they should appear in the constructed function names. **parasail** Required. The function prefix to avoid clashing with other libraries. All functions begin with this prefix. **(nw, sg, sw)** Required. The class of alignment; global, semi-global, or local, respectively, **stats** Optional. Return alignment statistics. **(table, rowcol)** Optional. Return the entire dynamic programming table or return the last row and column of the dynamic programming table, respectively. **(striped, scan, diag)** Optional. The vectorized approach. Striped is almost always the best choice. **profile** Vector approaches only, specifically only prefix scan and striped, but optional in that case. The prefix scan and striped vector implementations must first compute a query profile. That step can optionally be performed ahead of time and the query profile reused to avoid costly recomputation of the query profile. **(sse2_128, sse41_128, avx2_256, knc_512)** Vector approaches only, but optional in that case. The instruction set and vector width. If not given, the default will use CPU dispatching to select the best available instruction set on the host system. **(8, 16, 32, 64, sat)** Vector approaches only, but required in that case. The integer width of the solution. This also affects performance; a smaller integer width will increase the number of lanes used by the vectors and thus improve performance. If “sat” is used, the function defaults to the 8-bit implementation and, if overflow of the score is detected, it will retry using the 16-bit implementation.

The two examples to follow should help illustrate how function names should be constructed. **parasail_sw** As simple as possible, this is the non-vectorized reference implementation of Smith-Waterman local alignment. It will return the alignment score as well as the ending locations of the alignment along the query and database sequences. **parasail_sg_stats_scan_avx2_256_sat** The prefix scan vector implementation of semi-global alignment. It will use 8-bit integers during the computation and recompute using 16-bit integers if overflow is detected (‘sat’ is for ‘saturation’). AVX2 is selected for the CPU instruction set. Additional alignment statistics are also computed and returned.

### Instruction sets and CPU dispatching

Parasail supports the SSE2, SSE4.1, AVX2, and KNC (Xeon Phi) instruction sets. In many cases, a compiler can compile source code for an instruction set that is not supported by the host CPU. The code is still compiled, however, Parasail uses a technique called CPU dispatching to avoid running code that uses instructions that the host CPU does not support. CPU dispatching tests for features of the CPU at runtime which is different than testing whether a compiler can compile certain instructions at compile-time. The results of the runtime CPU tests are used to correctly select the appropriate Parasail implementation for the highest level of instruction set supported on the host platform. This allows Parasail to be compiled and distributed by a maintainer for the best available system while still allowing the same distribution to run with a lesser CPU.

Parasail is optimized for the SSE4.1 level of instruction set. Some SSE4.1 instructions are missing for SSE2 and were emulated using other available SSE2 instructions. Some AVX2 instructions, e.g. bit packing, lane shifting, also needed to be emulated using other AVX2 instructions because AVX2 is effectively two SSE 128-bit lanes operating independently and some operations do not cross the lane boundaries.

### Improvements to striped vectorization

Farrar’s striped method [7] remains the fastest Smith-Waterman inter-task algorithm to date. However, its implementation has seen two improvements. First, the SWPS3 [16] implementation improved the *lazy F evaluation loop*. This improvement was duplicated in the SSW [9] implementation.

More recently, the striped method was evaluated with respect to the number of vector lanes utilized within the calculation. For example, SSE uses a 128-bit vector that can be divided into 64-, 32-, 16-, or 8-bit integers which corresponds to two, four, eight, or sixteen vector lanes, respectively. A feature of the striped method is the recalculation of a column within the dynamic programming table until the column converges. Evaluating SSW showed that the number of corrective passes increased as the number of vector lanes was increased [13].

This problem is improved within Parasail using a technique of shifting the origin of the calculation towards the smallest representable integer. This takes advantage of the fact Smith-Waterman does not allow for negative values. For example, for 8-bit integers −127 is treated as 0, allowing the entire range of 0 through 255 to be utilized. Further, using saturation arithmetic keeps the calculation from underflowing. This is not unlike previous SSE2 implementations that mitigated the lack of a signed 8-bit vector maximum instruction and instead used unsigned integers [7]. However, there is not a corresponding unsigned 16-bit vector maximum instruction for SSE2 while there are sufficient signed saturation arithmetic operations available to perform the calculation. The combination of shifting the values and using saturation arithmetic significantly reduces the number of corrective passes needed for each column in the dynamic programming table.

Unfortunately, similar improvements were not possible for Needleman-Wunsch or semi-global alignment because the table values are allowed to be negative. As a result, in some cases the prefix scan implementation of those algorithms will perform better than the striped implementation [13].

One last improvement was made to the implementation of Zhao et al. [9]. In their implementation of local alignment, a copy of the entire column is made for the column containing the highest score. Parasail’s implementation avoids the costly memory copy by swapping pointer references to an auxiliary column. This only applies to local alignments; the global and semi-global implementations do not require the additional column.

### Code generation

The Parasail library uses a code generation step in addition to some compiler preprocessor directives to create the thousands of functions implemented within the library. This takes advantage of the observation that the basic algorithms for the three classes of alignments remain the same no matter which instruction set and integer width is used. End users do not need to worry about this code generation step, though for code development this has a number of benefits including faster time to solution when modifying the code as well as enforcing consistency between the myriad implementations possible.

### Verification

The Parasail library is rigorously tested using cross-verification to verify correct implementations of the various library functions. Given a test dataset in FASTA format, the result of the reference implementation for a given class of alignment is compared against all other implementations for the same class of alignment. This is done both for the resulting alignment scores as well as for the byte by byte comparisons of the entire dynamic programming tables involved. The software is not released until all tests pass for reasonably sized test datasets.

### Parasail aligner application

In addition to the Parasail library, the software also provides the *parasail_aligner* application. The aligner application will take a FASTA- or FASTQ-formatted query file as input and align all of the query sequences against a set of database sequences found in a second FASTA- FASTQ-formatted database file. Alternatively, if only one file is supplied, all of the sequences in the file will be compared against themselves. Since the Parasail library implements many different alignment routines, the name of the function to use must be specified to the aligner. Optionally, a filter can be applied to skip the alignment of any two sequences that don’t contain an exact-matching seed of the given length. This filter uses an enhanced suffix array data structure [17, 18] that allows for arbitrarily long exact-matching seeds.

## Results

The following benchmarks repeat those performed by Rognes [8]. The reader is encouraged to refer to Rognes’s original manuscript. The database sequences, accession numbers, score matrices and gap penalties are identical to those used previously, however they are repeated in later sections for convenience. Further, the figures and tables are intentionally similar in color, layout, and styling in order to more easily compare to the previous evaluation. However, the tests performed here do differ with respect to the software selected for evaluation. BLAST [4], BLAST+ [19], and SWIPE [8] were previously evaluated; the latest available versions were selected for the current evaluation. SWPS3 [16] is not evaluated. The latest implementation of Farrar’s striped method [7] is evaluated in the context of the SSW library [9]. The new implementation of the striped method within Parasail’s library is evaluated as “Parasail”. No previous evaluation of global and semi-global alignment performance has been performed. Since opal [14] and libssa [15] support global alignments, their performance is compared against Parasail’s implementations. When comparing the performance of various approaches and implementations, speed is reported in billion (giga) cell updates per second (GCUPS), where a cell is a value in the dynamic programming table.

Great care was taken to compare the various software libraries and applications fairly. All tests were run three times and the average GCUPS performance was recorded. The loading times for the database sequences were excluded from this study in order to focus on the algorithmic performance of the alignment routines. The following software reported the GCUPS performance directly for the alignments performed: Parasail, opal, SWIPE, and SSW. For the remaining software including BLAST, BLAST+, and libssa, a wall clock timer was patched into the code that reported on the alignment times only, and the wall clock time was used to calculate the GCUPS result based on the known amount of work for each test case performed.

### Software

Table 1 lists the software packages that were evaluated, including their version numbers and command line options used. The SSW library provides its own test program for performing alignments, but it was intentionally not used for benchmarking due to its additional overhead. Instead, the parasail_aligner was duplicated and modified to use the routines within the SSW library. Though opal and libssa are both software libraries and not stand-alone tools, they both provide an example application in addition to the library. The example applications are evaluated here, namely ‘opal_aligner’ and ‘libssa_example’. Opal’s aligner was only available as a single-threaded application.

**Table 1:** Software included in performance testing

Software	Version	Command Line
BLAST	2.2.26	blastall -p blastp -F F -C 0 -b 0 -v 10 -a $T -M $M -G $O -E $E -i $Q -d $D
BLAST+	2.2.31+	blastp -seg no -comp_based_stats F -num_alignments 0 -num_descriptions 10 -num_threads $T -matrix $M -gapopen $O -gapextend $E -query $Q -db $D
SWIPE	2.0.11	swipe -v 10 -a $T -M $M -G $O -E $E -i $Q -d $D
libssa	29 October 2015	libssa_example -c 10 -N $T -M $M -O $O -E $E -t SW -b 16 -s AVX2 -i $Q -d $D
opal	16 November 2015	opal_aligner -x 1 -a SW -o $O -e $E -m $M -s $Q $D
SSW	30 July 2015	test_ssw -x -t $T -o $O -e $E -m $M -f $D -q $Q
Parasail	12 August 2015	parasail_aligner -x -a $A -t $T -o $O -e $E -m $M -f $D -q $Q

Command line variables: threads ($T), score matrix file name ($M), gap open ($O) and extension ($E) penalties (positive values), query file name ($Q), database file basename ($D), Parasail alignment function name ($A)

The parasail_aligner can be run using any one of the many alignment routines the Parasail library provides. The initial benchmarks compare other local alignment implementations against Parasail’s local alignment implementation. The latter benchmarks compare Parasail’s local alignment performance against its global and semi-global performance for the striped and scan vectorized approaches. The following Parasail functions were evaluated.

- parasail_sw_striped_profile_sse41_128_sat,
- parasail_sw_striped_profile_avx2_256_sat,
- parasail_nw_striped_profile_sse41_128_16,
- parasail_nw_striped_profile_avx2_256_16,
- parasail_nw_scan_profile_sse41_128_16,
- parasail_nw_scan_profile_avx2_256_16,
- parasail_sg_striped_profile_sse41_128_16,
- parasail_sg_striped_profile_avx2_256_16,
- parasail_sg_scan_profile_sse41_128_16, and
- parasail_sg_scan_profile_avx2_256_16.

### Hardware

Results were taken on compute nodes of the Constance cluster, part of Pacific Northwest National Laboratory’s Institutional Computing. Each Constance node contains dual Intel Haswell E5-2670 CPUs (2.3 Ghz) giving 24 cores and 64 GB 2133Mhz DDR4 memory per node. Intel’s Haswell CPUs support the AVX2 instruction set. Although these processors are capable of hyper-threading, which would have given each node 48 logical cores, it was not used for these experiments. Hyper-threading is intentionally disabled as a matter of policy because Constance is a general use cluster and hyper-threading benefits are strongly application dependent [20]. Because it was not enabled, only up to 24 cores were utilized.

### Database and query sequences

The UniProt Knowledgebase Release 11.0 [21] (consisting of both Swiss-Prot release 53.0 and TrEMBL release 36.0) was chosen because it duplicates the benchmark evaluation performed by Rognes in [8]. The dataset was originally chosen for being a realistic dataset less than 2GB in size because some of the software originally tested would fail for larger file sizes. The current software tested does not have the same input size limits, however the same dataset is used for consistency with the previous evaluation. This validates Rognes’s original intent of selecting a dataset that should be available for download in the foreseeable future, since the new evaluation presented here occurs over eight years later.

The query sequences used here are identical to the ones used by Rognes. The 32 accession numbers are [Swiss-Prot:P56980, Swiss-Prot:O29181, Swiss-Prot: P03630, Swiss-Prot:P02232, Swiss-Prot:P01111, Swiss-Prot: P05013, Swiss-Prot:P14942, Swiss-Prot:P00762, Swiss-Prot:P53765, Swiss-Prot:Q8ZGB4, Swiss-Prot: P03989, Swiss-Prot:P07327, Swiss-Prot:P01008, Swiss-Prot:P10635, Swiss-Prot:P58229, Swiss-Prot:P25705, Swiss-Prot:P03435, Swiss-Prot:P42357, Swiss-Prot: P21177, Swiss-Prot:Q8LLD0, Swiss-Prot:O60341, Swiss-Prot:P27895, Swiss-Prot:P07756, Swiss-Prot:P04775, Swiss-Prot:P19096, Swiss-Prot:P28167, Swiss-Prot: P0C6B8, Swiss-Prot:P20930, Swiss-Prot:P08519, Swiss- Prot:Q7TMA5, Swiss-Prot:P33450 and Swiss-Prot: Q9UKN1]. Note that [Swiss-Prot:Q8LLD0] replaces [Swiss-Prot:Q38941]. The queries range in length from 24 to 5478 residues. As done previously by Rognes, some of the tests were only performed with the 375 residues long P07327 query, representing a protein of roughly average length [8].

### Score matrices and gap penalties

The score matrices and gap penalties selected for this evaluation duplicate those used by Rognes [8] so that a direct comparison can be made between the two evaluations. Previously, 82 different combinations of amino acid substitution score matrices and gap penalties accepted by BLAST were tested, including BLOSUM45, BLOSUM50, BLOSUM62, BLOSUM80, and BLOSUM90 from the BLOSUM series [1] as well as PAM30, PAM70, and PAM250 from the PAM series [2]. These matrices previously represented the ones supported by earlier BLAST versions, though there are currently 84 combinations of amino acid substitution score matrices and gap penalties accepted by the ‘blastp’ website [22]. Matrices were obtained from the NCBI FTP site. Again, duplicating the same evaluation criteria as Rognes, some of the tests were only performed with the BLOSUM62 matrix and gap open and extension penalties of 11 and 1, respectively, which is the BLAST default [8].

### Threading evaluation

**Fig. 1:**
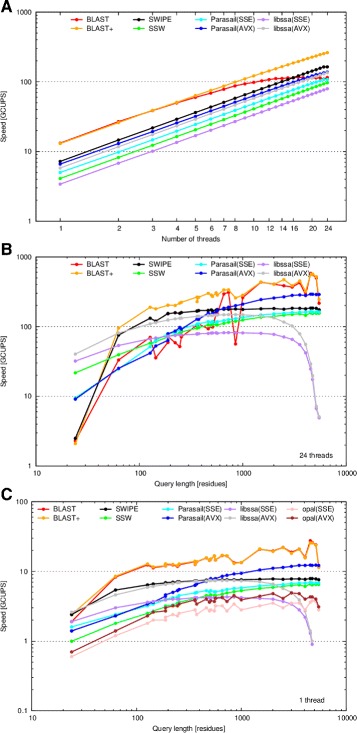
Performance dependency on number of threads and query length. The speed in billion cell updates per second (GCUPS) of BLAST (*red*), BLAST+ (*orange*), SWIPE (*black*), SSW (*green*), Parasail’s striped SW implementation using SSE4.1 (*light blue*) and AVX2 (*dark blue*) instruction sets, as well as libssa using SSE4.1 (*purple*) and AVX2 (*gray*), using a variable number of threads and queries of varying length. Opal is only evaluated in **c** since the application was single-threaded (SSE4.1 as pink, AVX2 as brown). **a** Number of threads ranging from 1 to 24 and the 375 residue long P07327 query sequence. **b** Query sequences ranging from 24 to 5478 residues in length and 24 threads. **c** Query sequences of varying length and 1 thread. All scales are logarithmic. The BLOSUM62 matrix and gap open and extension penalties of 11 and 1, respectively, were used in all cases. This figure corresponds to Figure 6 in Rognes [8]

Figure 1a shows the performance characteristics of all software as the number of threads increase from 1 to 24. The query sequence was fixed at the 375 residue [Swiss-Prot:P07327]. Additionally, the BLOSUM62 matrix and gap open and extension penalties of 11 and 1 were used.

Compared to the previous evaluation in [8], the performance of all striped implementations perform significantly better than previously reported. At best, striped (SSW) had a performance range of 3.7 to 15.0 GCUPS and now runs from 4.1 to 96.6 GCUPS. This large difference is attributed to both improved sequence database processing as well as the use of better workload scheduling. The performance of Parasail’s SSE4.1 implementation ranges from 5.0 to 107.5 GCUPS while the AVX2 implementation ranges from 6.6 to 137.7 GCUPS. SWIPE previously ran from 9.1 to 106.2 GCUPS and now runs from 7.2 to 163.6 GCUPS, indicating a relatively small drop in performance for a single thread while significantly improving on multithreaded performance. libssa’s SSE4.1 implementation ranges from 3.4 to 79.4 GCUPS while the AVX2 implementation ranges from 5.8 to 135.1 GCUPS. BLAST reaches an early peak of 116.5 GCUPS at 13 threads while BLAST+ continues to scale to upwards of 261.8 GCUPS. Overall, BLAST+ performs the best while the striped implementations are narrowly below SWIPE in performance.

### Query length evaluation

Figure 1b and c show the performance characteristics of all software as the query lengths vary, while keeping the number of threads fixed to 24 threads (B) or one thread (C). Additionally, the BLOSUM62 matrix and gap open and extension penalties of 11 and 1 were used. The query lengths ranged from 24 to 5,478 amino acid residues.

Using 24 threads, similar to the threading evaluation above, the performance of all striped implementations perform significantly better than previously reported. SSW ranged from 21.7 to 156.9 GCUPS while previously ranging from 1.2 to 46.6 GCUPS. The Parasail implementations start off somewhat slower at 9.5 and 9.1, and peak at 164.1 and 291.5 for SSE4.1 and AVX2, respectively. Generally, longer queries performed better for all software evaluated. SWIPE was an exception, having a rather flat performance curve but quickly maxing out at 184.0 GCUPS. libssa was the other exception, having peaked at 82.0 and 148.4 GCUPS for SSE4.1 and AVX2, respectively, but performance quickly dropped off for sequences longer than 1,000 amino acids. Parasail’s AVX2 implementation begins to outperform SWIPE for query sequences longer than approximately 500 amino acids. BLAST+ again was the strongest performer, topping out at 5654.9 GCUPS.

Using a single thread compared to all 24 threads, similar performance characteristics are noted. Performance peaks of 7.9, 6.6, 6.9, 12.3, 4.3, and 7.4 GCUPS are noted for SWIPE, SSW, Parasail SSE4.1, Parasail AVX2, libssa SSE4.1, and libssa AVX2, respectively. BLAST and BLAST+ perform similarly and peak near 27 GCUPS. Opal is evaluated using a single thread, peaking at 3.8 and 4.9 GCUPS for its SSE4.1 and AVX2 implementations, respectively, the slowest of any implementation evaluated.

### Scoring system evaluation

**Fig. 2:**
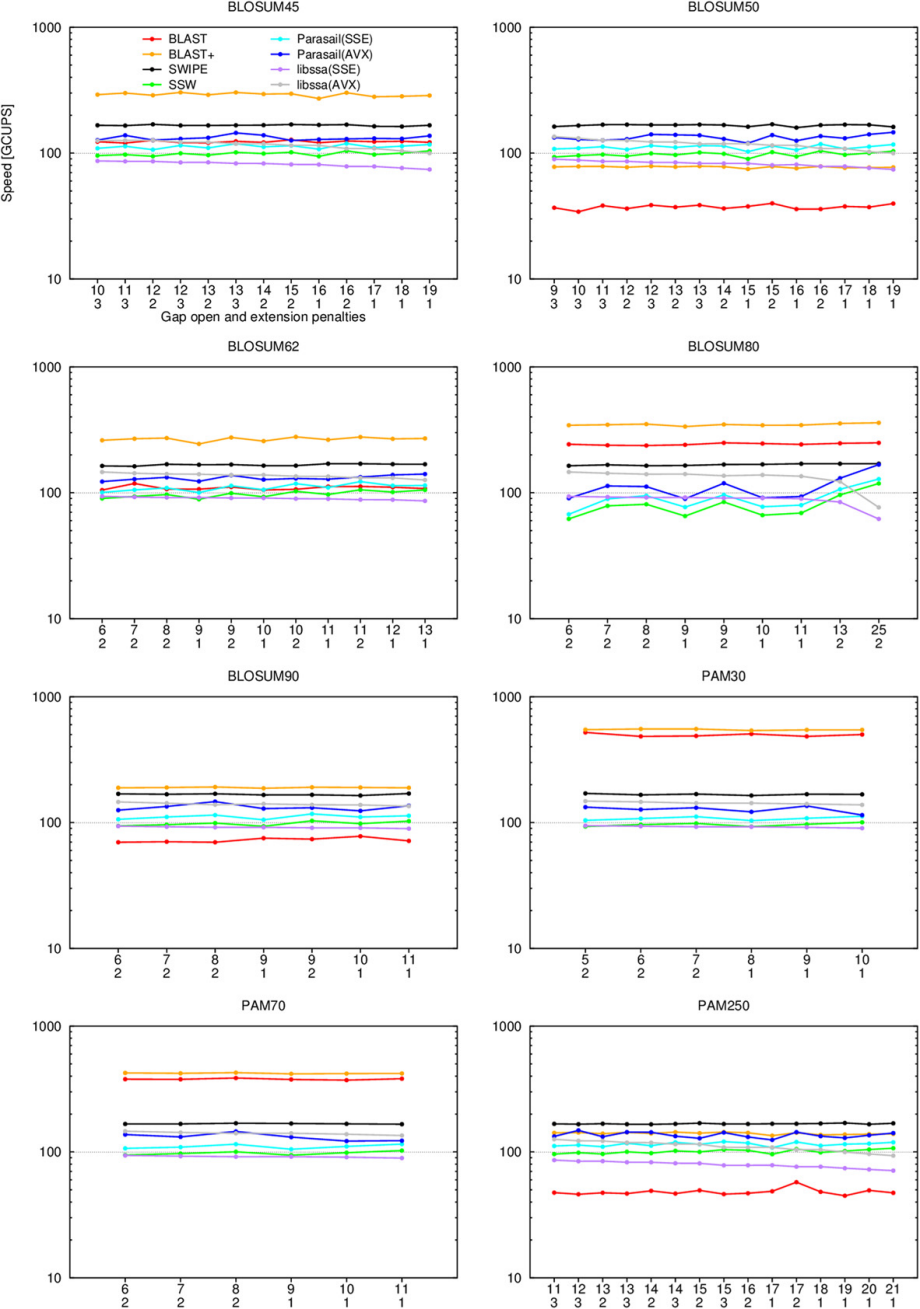
Performance with different scoring systems. The speed in billion cell updates per second (GCUPS) (logarithmic scale) is shown for BLAST (*red*), BLAST+ (*orange*), SWIPE (*black*), and SSW (*green*), Parasail’s striped SW implementation using SSE4.1 (*light blue*) and AVX2 (*dark blue*) instruction sets, as well as libssa using SSE4.1 (*purple*) and AVX2 (*gray*), using different scoring systems. All combinations of scoring matrices and gap penalties accepted by BLAST were tested. The matrix name is indicated above each graph, while the open and extension gap penalties are indicated on the x-axis. The query sequence was P07327 and 24 threads were running. This figure corresponds to Figure 7 in Rognes [8]

Figure 2 shows the performance characteristics of all software as the scoring conditions are varied. All 82 combinations of matrices and gap penalties previously evaluated by Rognes [8] are repeated here. The 375 residue long P07327 query sequence and 24 threads were used.

The striped implementations again perform better than previously reported. Previously striped was running at an almost constant 14 GCUPS while SSW now shows a dependence on the scoring criteria with an average of 96.5±9.0 GCUPS. Both Parasail implementations show a similar dependence on the scoring criteria of 109.5±10.0 GCUPS and 130.4±12.4 GCUPS for SSE4.1 and AVX2, respectively. SWIPE runs better at 166.9±2.3 GCUPS compared to the previous 104±2 GCUPS. libssa performs better than its SWIPE counterpart when using AVX2 instructions, running at 125.7±15.5 GCUPS, while performing only at 85.5±6.7 GCUPS using SSE4.1 instructions. The performance of BLAST and BLAST+ was highly dependent on the scoring matrix used, with GCUPS of 146.8±137.5 and 247.1±134.9, respectively.

### Evaluation of global and semi-global implementations

The threading and query length evaluations were additionally performed using global and semi-global alignment routines.

libssa’s global alignment capabilities were evaluated. Due to the lack of an available multithreaded application, opal’s global alignment evaluation was limited to the lone single-threaded evaluation. In addition to Parasail’s striped vector approach, the prefix scan approach is also evaluated. Unlike in the evaluation of local alignments where the 8-bit saturation-checking routines were used, the 16-bit element versions of the global and semi-global alignment routines were used because the 8-bit versions almost always saturated, resulting in poor performance with wasted computation. This was true both for Parasail as well as libssa.

**Fig. 3:**
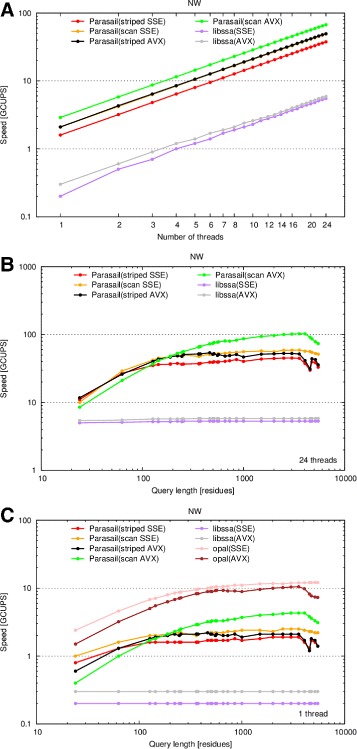
Performance dependency on number of threads and query length for global alignments. The speed in billion cell updates per second (GCUPS) of striped SSE4.1 vectors (*red*), prefix scan SSE4.1 vectors (*orange*), striped AVX2 vectors (black), and prefix scan AVX2 vectors (*green*), as well as libssa using SSE4.1 (*purple*) and AVX2 (*gray*) instruction sets, using a variable number of threads and queries of varying length. **a** Number of threads ranging from 1 to 24 and the 375 residue long P07327 query sequence. **b** Query sequences ranging from 24 to 5478 residues in length and 24 threads. **c** Query sequences of varying length and 1 thread. All scales are logarithmic. The BLOSUM62 matrix and gap open and extension penalties of 11 and 1, respectively, were used in all cases

**Fig. 4:**
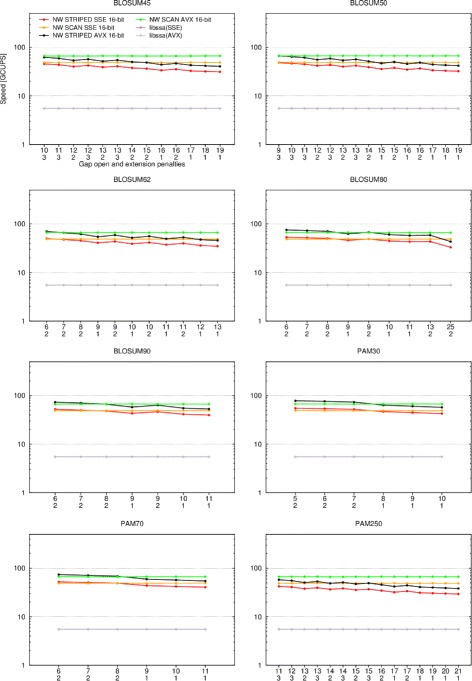
Performance with different scoring systems for global alignments. The speed in billion cell updates per second (GCUPS) (logarithmic scale) is shown for striped SSE4.1 vectors (*red*), prefix scan SSE4.1 vectors (*orange*), striped AVX2 vectors (black), and prefix scan AVX2 vectors (*green*), as well as libssa using SSE4.1 (*purple*) and AVX2 (*gray*), using different scoring systems. Note that the libssa results completely overlap. All combinations of scoring matrices and gap penalties accepted by BLAST were tested. The matrix name is indicated above each graph, while the open and extension gap penalties are indicated on the x-axis. The query sequence was P07327 and 24 threads were running

Figure 3 evaluates the threading performance while Fig. 4 evaluates the scoring system of global alignment routines. Parasail’s prefix scan implementation outperforms the implementation of Farrar’s striped approach. Though not shown in Figs. 1 and 2, the prefix scan implementation only outperforms the striped implementation for global alignments; striped is faster for local as well as semi-global alignments. This is attributed to the higher number of corrective passes that must be made during the striped computation for global alignments [13].

Unfortunately, libssa is not performing as expected for global alignments. libssa was previously reported to be nearly two times faster than opal [15], though here opal is outperforming all other implementations by a wide margin for single-threaded global alignments. Parasail is also outperforming libssa for all alignments evaluated. Examining the output of these runs showed that libssa was detecting overflow of the 16-bit calculations for all query and database sequence combinations, forcing libssa to perform a 64-bit evaluation instead. This is clearly an error, since Parasail is not detecting overflow during its 16-bit calculations. This unfortunate result is attributed to libssa being research code; the results presented in [15] validate the approach even though the current evaluation was unable to reproduce favorable results.

Semi-global results are similar to global results for opal. libssa does not implement semi-global alignment and as such is not evaluated. For Parasail’s striped implementation, the semi-global routines are slightly faster than the global routines but significantly slower than local alignments. Parasail’s scan implementation is slower than the striped implementation for semi-global alignments.

For Parasail’s prefix scan implementations, each column of the dynamic programming table is iterated over twice. This leads to stable, predictable performance of the prefix scan vector approach. The prefix scan routines have stable performance for the global classes of alignments. This stable performance of the prefix scan vector routines is especially evident in Fig. 4. Even considering the stable performance of the prefix scan routines, the striped approach is always faster for local alignments and semi-global alignments.

### Discussion

The Parasail library is an improvement over earlier SIMD intra-sequence implementations. The performance reported here is also better than what was previously reported for intra-sequence alignments [8].

The intent of the Parasail library is to be integrated into other software packages, not necessarily to replace the already highly performing database search tools such as BLAST [4], SWIPE [8], or libssa [15]; database search on its own is an important problem with satisfactory solutions. As a software library that focuses on individual pairwise alignments, Parasail can be more readily adapted into other software packages needing such a capability, such as those described by [9] or as part of other programming language packages such as scikit-bio [23].

The Parasail library represents the first time global, semi-global, and local alignment routines are available as a high performance software library. The routines have been written to utilize the latest x86 CPU instruction sets while remaining compatible with any platform. The modular implementation and code generation process will easily allow Parasail to be adapted to future instruction sets with wider vector units as they become available.

Future versions of Parasail will add the capability of returning alignment tracebacks. Though Parasail already has the option of returning additional alignment statistics, the full traceback is important in some cases. As open source software, the intent is to welcome feature requests, enhancements, and fixes from the growing Parasail community.

## Conclusions

The Parasail library is an improvement over earlier SIMD intra-sequence implementations. Applications that require optimal alignment scores could benefit from the improved performance. For the first time, SIMD global, semi-global, and local alignments are available in a stand-alone high-performance C library.

## Availability and requirements

**Project name:** Parasail - Pairwise Sequence Alignment Library**Project home page:**https://github.com/jeffdaily/parasail**Operating system(s):** Platform independent**Programming language:** C, with Python language bindings**Other requirements:** SSE2, SSE4.1, and/or AVX2 compiler intrinsics preferred**License:** Battelle BSD-style**Any restrictions to use by non-academics:** N/A

## Additional file

Additional file 1
**Parasail v1.0.0 source code.** The source code of Parasail version 1.0.0 as a gzipped tar archive file. (GZ 1180 kb)

## Abbreviations

GCUPS: giga cell updates per second
SIMD: single instruction multiple data
SSE,SSE2,SSE4.1: streaming SIMD extensions, version 2, or version 4.1
AVX,AVX2: advanced vector extensions, version 2
SW: Smith-Waterman local alignment
NW: Needleman-Wunsch global alignment
SG: semi-global alignment
