# Supplementary figures and images for: Parasail: SIMD C library for global, semi-global, and local pairwise sequence alignments
Source: BMC Bioinformatics. 2016 Feb 10;17:81. doi: 10.1186/s12859-016-0930-z (PMC4748600; doi:10.1186/s12859-016-0930-z)

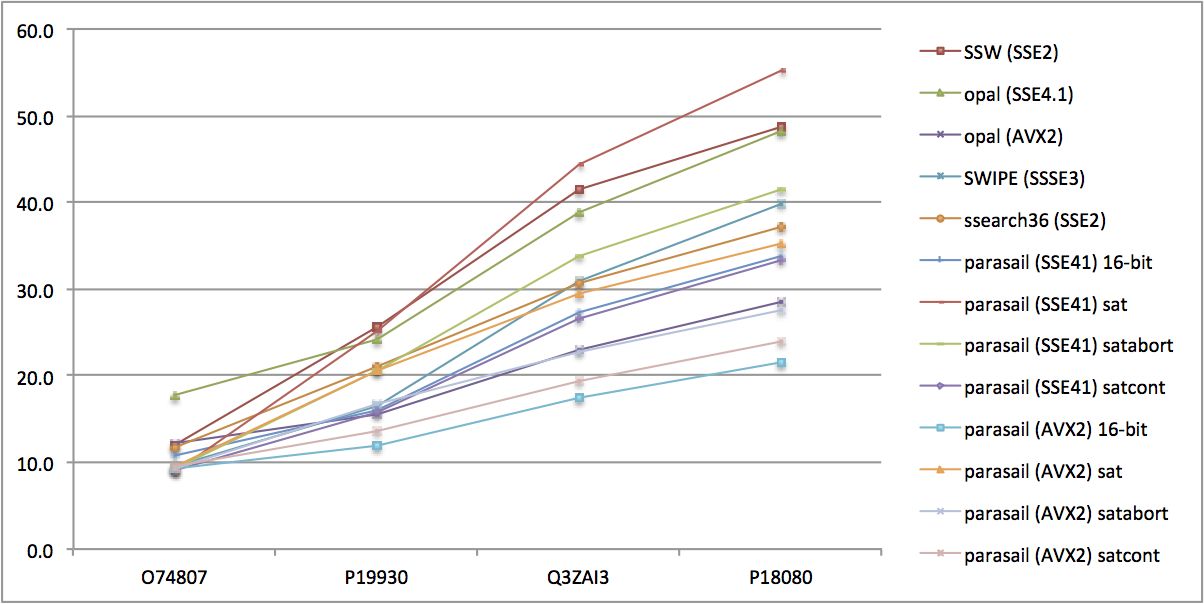

Supplement: Additional file 1 — Parasail v1.0.0 source code. The source code of Parasail version 1.0.0 as a gzipped tar archive file. (GZ 1180 kb) [file 12859_2016_930_MOESM1_ESM.gz › parasail-1.0.0/images/perf_haswell.png]

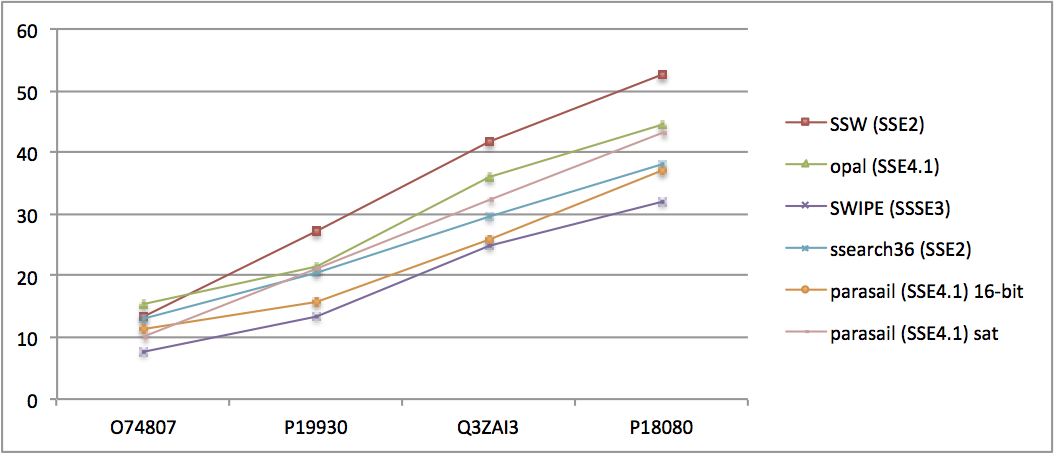

Supplement: Additional file 1 — Parasail v1.0.0 source code. The source code of Parasail version 1.0.0 as a gzipped tar archive file. (GZ 1180 kb) [file 12859_2016_930_MOESM1_ESM.gz › parasail-1.0.0/images/perf_mac.png]
